# Seroepidemiology of Bovine Viral Diarrhoea Virus (BVDV) in the Adamawa Region of Cameroon and Use of the SPOT Test to Identify Herds with PI Calves

**DOI:** 10.1371/journal.pone.0021620

**Published:** 2011-07-06

**Authors:** Ian G. Handel, Kim Willoughby, Fiona Land, Bronwyn Koterwas, Kenton L. Morgan, Vincent N. Tanya, Barend M. deC. Bronsvoort

**Affiliations:** 1 The Roslin Institute at the Royal (Dick) School of Veterinary Studies, University of Edinburgh, Easter Bush, Midlothian, United Kingdom; 2 Department of Veterinary Clinical Sciences, University of Liverpool, Leahurst Veterinary Teaching Hospital, Neston, Wirral, United Kingdom; 3 Ministry of Scientific Research and Innovation, Yaounde, Cameroon; 4 Moredun Research Institute, Pentlands Science Park, Bush Loan, Penicuik, Midlothian, United Kingdom; Instituto Butantan, Brazil

## Abstract

Bovine viral diarrhoea, caused by the bovine viral diarrhoea virus (BVDV) in the Pestivirus genus of the Flaviviridae, is one of the most important diseases of cattle world wide causing poor reproductive performance in adult cattle and mucosal disease in calves. In addition it causes immunosuppression and increased susceptibility to other infections, the impact of which is uncertain, particularly in sub-Saharan Africa where animals are exposed to a much wider range and higher intensity of infections compared to Europe. There are no previous estimates of the seroprevalence of BVDV in cattle in Cameroon. This paper describes the serological screening for antibodies to BVDV and antigen of BVDV in a cattle population in the Adamawa Region of Cameroon in 2000. The estimates of herd-level and within herd seroprevalences adjusted for test imperfections were 92% and 30% respectively and 16.5% of herds were classed as having a persistently infected calf (PI) in the herd within the last year based on the “spot” test approach. There was evidence of clustering of herds with PI calves across the north and west of the Region which corresponds with the higher cattle density areas and of self-clearance of infection from herds. A multivariable model was developed for the risk of having a PI calf in the herd; proximity to antelope, owning a goat, mixing with 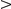
10 other herds at grazing and the catchment area of the veterinary centre the herd was registered at were all significant risk factors. Very little is known about BVDV in sub-Saharan Africa and these high seroprevalences suggest that there is a large problem which may be having both direct impacts on fertility and neonate mortality and morbidity and also indirect effects through immunosuppression and susceptibility to other infections. Understanding and accounting for BVDV should be an important component of epidemiological studies of other diseases in sub-Saharan Africa.

## Introduction

Bovine viral diarrhoea virus (BVDV) is a single stranded RNA virus belonging to the *Pestivirus* genus of the family *Flaviviridae*. BVD is widespread and economically one of the most important diseases of cattle. It can result in poor reproductive performance, reduced milk yield, ill thrift and immunosuppression. Clinically, there are three forms of the infection: (1) A persistently infected form (PI); (2) an acute transient form, which may be subclinical or may present as fever, diarrhoea and short-term immunosuppression which may complicate other diseases such as pneumonia[1]; and (3) mucosal disease characterised by severe erosive lesions in the oral and intestinal mucosa, diarrhoea and death[2], which only occurs in PI animals. In the pregnant animal the virus can be transmitted vertically to the foetus. The timing of transmission is critical to the epidemiology of the disease; infection prior to 100–125 days of gestation may result in foetal death or in birth of a live calf persistently infected with BVDV, due to the infection occurring prior to the development of immune competence[3]. Such PI calves are born immunotolerant and remain persistently viraemic for their entire life. They may appear stunted and fail to thrive, or may appear clinically normal, allowing them to remain in a herd or be mixed with other herds without knowledge of their infective status. PI calves have high mortality rates and many succumb to mucosal disease between six months and two years of age[2] although little is known about how long they may live in an African setting. PI animals are the most important source of infection to other animals through nose to nose contact[4] and for the maintenance of the virus in the population. Their identification and removal is considered key to control efforts [5]. Several studies have demonstrated significant seroprevalence in African wildlife, although the role of sheep and wildlife in BVDV transmission is yet to be fully elucidated[6]–[8]. Once a PI animal enters a herd, it has been assumed that the herd is likely to remain infected due to the ongoing production of PI animals as naïve cows are infected during pregnancy, however, recent evidence suggests that herds may self-clear [9].

Reported seroprevalence of BVDV varies between different regions. There is evidence for infection in several African countries, although there do not appear to be any previous reports of the virus from Cameroon. It has been reported from a number of other African countries including South Africa [10], Zambia [11] and Ivory coast[12]. However there appears to be little recent data for Africa and in particular nothing on the molecular types circulating in the region. Following transient infection animals will remain antibody positive for life, so antibody prevalence reflects the proportion of animals previously exposed to BVDV at any point in life [2].

The Adamawa Region is a high plateau region of Cameroon stretching across middle of the country and is one the main cattle rearing zones. The husbandry practices vary within the Region but in general cattle are managed in herds of 50–70 animals by a single herdsman. Each dry season 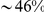 of herdsmen will split their herds and send a portion on transhuamnce to find grazing along the wetter river valleys, spreading the risk of drought and lack of grazing in the main home area. Most herds (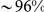) are grazed on communal land and between 15–40% of herds are fenced at night. Contacts with other herds vary according to husbandry activities but on an average day on transhumance a herd might contact 7–10 other herds compared to 4–6 at the home pasture and 1–3 at the watering points[13]. There was no licence to import BVDV vaccines at the time of the study and herdsmen did not report using any vaccines other than those required by the Cameroon government.

This paper describes the results of a population-based cross sectional study of the seroprevalence of BVDV in the Adamawa Region of Cameroon based on archived sera collected in 2000. The study aimed to characterise the geographical and epidemiological picture of BVDV infection in the region, estimate the herd-level, PI and within-herd seroprevalences and identify risk factors for being a herd with a PI calf and for self-clearance. In order to incorporate the structure of the original study design, the seroprevalence analysis used multi-level modelling approaches and a Bayesian framework was used to allow the incorporation of test imperfections and the sampling design in the estimation process[14].

## Methods

### Ethical Statement

This study used cattle sera biobanked in 2000. The cattle were sampled by a qualified veterinary surgeon with the consent of the animal owner and in accordance with the Cameroonian Ministry of Research (MINREST) guidelines and approval from the University of Liverpool ethics committee in 1999.

### Samples

**Figure 1:**
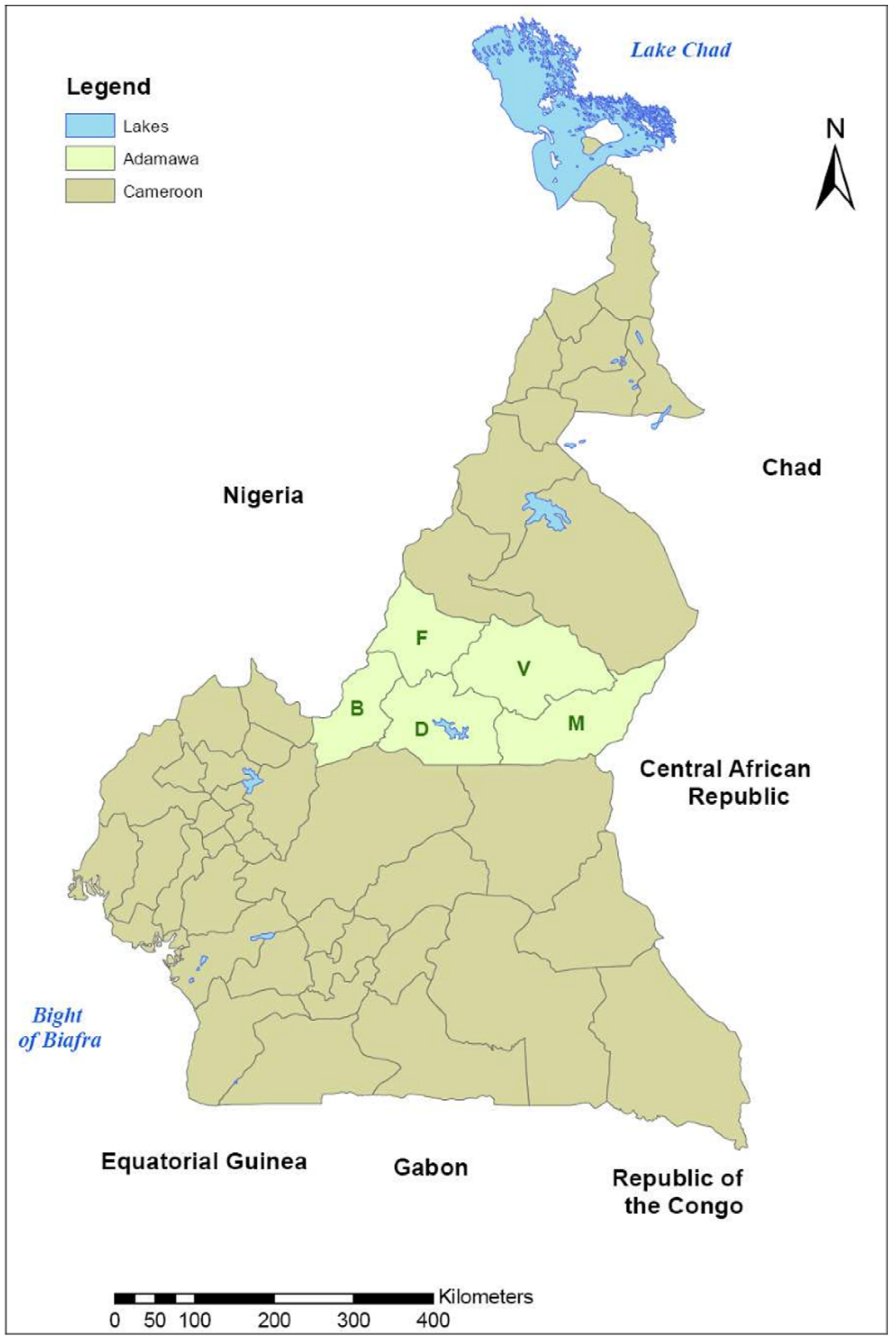
Political map of Cameroon showing the Adamawa Region and the five administrative Regions within it. (V = Vina; M = Mbere; D = Djerem; B = Mayo Banyo; F = Faro et Deo).

The samples used for this investigation were originally collected as part of a study of foot-and-mouth disease (FMD). The study population has been described in detail [13]. Briefly, the study area was the Adamawa Region of Cameroon, an area of approximately 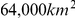 lying between latitudes 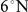 and 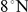 (Figure 1). It is divided into five administrative divisions (Vina, Mbere, Mayo Banyo, Djerem and Faro et Deo), with 88 Ministry of Livestock, Fisheries and Animal Industries (MINEPIA) veterinary centres distributed across it. A database of 13,006 herds constructed from rinderpest vaccination records was used as the sampling frame. A cross sectional study design was used and a stratified two stage random cluster sample was collected. The sampling was stratified by division and sampling was proportional to the number of herds registered in that division. Veterinary centres were randomly selected with replacement and with a probability proportional to the number of herds registered there. Three herds were then randomly sampled per selected veterinary centre without replacement (or multiples of three if a centre was selected more than once). The sample size was calculated on the basis of an assumed FMD herd seroprevalence of 50% [13] with 9% accuracy and 90% confidence with an additional 10% inflation to allow for refusals. Although not designed with BVD in mind this design was considered reasonable given the likely within-herd seroprevalences and endemic nature of this disease in Africa.

Herds were visited between April and October 2000. Samples were collected from 146 herds. Where available, a minimum of five adult (more than 24 months of age) and five juvenile (8 to 24 months of age) animals were randomly sampled from livestock presented by the herdsman using random number tables. The criteria of 8–24 months for classification as juvenile was selected to ensure that there was minimal misclassification due to the presence of maternal antibodies to FMDV, whilst capturing a reasonable growth period given that these cattle mature more slowly than their European counterparts. In several smaller herds fewer than five animals of the appropriate age class were available. In such cases all available animals in each age class were sampled giving a slightly smaller than expected sample of 1377 individual samples in total. The within herd sample size of five in each age strata was originally calculated to have a 95% probability of detecting at least 1 FMDV seropositive in a herd of 70 assuming a conservative within herd FMD seroprevalence of 50% and perfect test performance. The number of animals present in a herd was also recorded and ranged from 7 to 81 with a median of 35 and mean of 37.4. Blood was sampled by jugular venepuncture and allowed to clot. At the end of each day the blood samples were centrifuged in the field and approximately 3.5 ml of serum was separated from each and divided into two 1.8 ml cryovials (Nunc). The samples were kept at 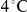 in a portable gas refrigerator until they could be frozen and stored at 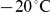, then transported to the UK on dry ice. They have since been stored at the FMD World Reference Laboratory (WRL), Pirbright, at 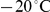.

### Questionnaire

A standardised questionnaire was used to collect information from herdsmen about herd management, movements, contact with other herds and wildlife. The questionnaire was completed at the time of blood sampling by an experienced interviewer in Foulfoulde and took 30–40 minutes. The design and administration of the questionnaire has been described in detail by Bronsvoort *et al*. [13]. All questionnaire and sample results were entered into an Access (Microsoft) database prior to analysis.

### Antibody ELISA

All 1377 serum samples were screened for antibodies to BVDV and other pestiviruses using a competitive ELISA (Institut Pourquier). There is cross reaction between other pestiviruses such as Border Disease and Classical Swine Fever on the test but the majority of seropositives are highly likely to be due to BVDV. The test was performed as per the manufacturer's instructions, except that test samples were run as single wells only due to cost limitations. The plates were then read on an automatic plate reader at 450 nm. The intensity of the colour (measured as optical density, OD) is an inverse measure of the concentration of anti-NS3 antibodies present in the test sample. The results are expressed as an inhibition percentage, calculated in equation (1).
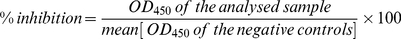
(1)


All serological testing was carried out blind. Samples were coded by herd and individual sample numbers so the operators were not aware of the animal's history. Plates were validated based on the criteria given by the manufacturer such that the negative controls had to have a minimum mean OD value of 0.8 and a percentage competition of 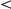
20% of the positives controls. Plates that failed this validation step were repeated. The point estimates for the Se and Sp of the test are stated as 97.4% and 97.2% respectively by the manufacturer.

**Antigen ELISA**

A subset of antibody negative samples from the NS3 antibody screening above were tested for BVDV NS 2–3 and 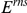 antigens using an antigen capture ELISA, (BVD/MD Mixed Antigen Screening ELISA, Institut Pourquier), performed as per the manufacturer's instructions. The plates were read on an automatic plate reader at 450 nm. The sample/positive control ratio (S/P) was calculated using equation 2 and samples were classified as negative/not PI with BVDV if the S/P ratio was 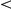
25; doubtful if it was 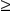
25 but 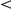
30; and positive/BVDV PI if the S/P ratio was 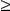
30.
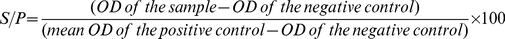
(2)


Plate results were considered valid if the mean OD of the positive controls reached at least a value of 0.8 and the ratio between the mean OD 450 value of the positive control and the OD 450 value of the negative control was greater than or equal to 4.5. Plates that failed this validation step were repeated.

The testing of antibody negative cattle for antigen was used to detect PI animals. Only juveniles were tested because PI animals are known to have increased mortality [15]. The likelihood of detecting transiently viraemic (ie. acutely infected) animals was low as viraemia generally only lasts four to ten days [3] as compared with the permanent viraemia in a PI animal. So although virus isolation from repeat samples was not carried out, for the purposes of this study the detection of any antibody negative/antigen positive animals was considered to represent PI detection.

### Estimating cattle density

Proxy data for cattle density was estimated based on the number of herds registered at each MINEPIA veterinary centre. The total cattle number for each veterinary centre was estimated as the product of the number of herds registered for the area and the mean herd size for all sampled herds. Voronoi tessellation was used to estimate the physical coverage area of each veterinary centre by constructing polygons where each point in the polygon is nearer to it's corresponding veterinary centre than any other veterinary centre. The tessellation was performed using the PBSMapping package in R [16]. The total cattle head estimate was then divided by the estimated physical coverage area of the veterinary centre to estimate cattle head per square kilometre.

### Estimating the last date a herd was exposed

The age-seropositivity status of the animals within a herd may be used to estimate the time since the herd last contained or was exposed to a persistently infected (PI) animal. Herds with young seropositive animals are likely to have been recently exposed, herds with only sero-negative young animals are likely to have been last exposed some time ago as transmission from non-PI animal to non-PI animal is rare[17]. To quantify this estimate we use a simple model of serological status. The test result for the 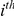 animal (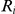) is modelled, with diagnostic test uncertainty, as a function of its true serological status (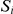), the test sensitivity (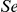) and the test specificity (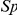):
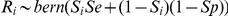
where 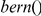 represents a single Bernoulli trial. Animals are considered to seroconvert with an unknown probability, 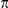 if exposed to a PI animal:
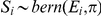



Animals are considered to have been exposed if they were born before the last date that a PI animal was present in the herd (i.e. 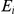 is an indicator variable set to 1 if the PI animal and 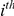 animal were ever alive at the same time and 0 otherwise). The time since the herd was last exposed to a PI animal was estimated using a Bayesian framework using MCMC simulations. Priors for the diagnostic test performance were as used in the previous section. A vague (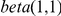) prior was used for 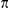, the probability that an exposed animal seroconverts and a 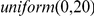 prior was used for the time since a last PI was present, implying that we expect PI animals to have been present at some time in the previous 20 years.

### Modelling within-herd seroprevalence

As the antibody ELISA has an imperfect sensitivity and specificity sero-prevalence was estimated using a statistical model based approach which explicitly corrects the estimates for the imperfection of the diagnostic test. The model was developed based on the framework used by Branscum *et al.* [14]. Counts of ELISA test positive animals, 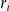, in the 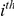 herd's sample were assumed to be distributed:
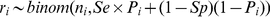
(3)where 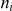 is the number of animals sampled, 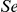 and 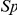 are the ELISA test sensitivity and specificity and 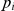 is the prevalence of sero-conversion. The within-herd prevalence, 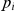 is assumed to be distributed as a zero-inflated mixture:
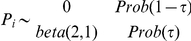
where 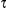 is the probability that any animal in the herd is seropositive. This effectively models the herds as having a seroprevalence of zero, i.e. unexposed or as exposed with a prevalence in the 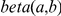 distribution. In the absence of other data, 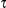 was given a vague prior distribution 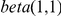. The within-herd prevalence of sero-positive herds was given an informative prior of 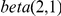. This has the effect of making herds with higher numbers of test positive juvenile animals more likely to be classified as genuinely seropositive in accordance with previous studies[17].

The prior distributions for the BVD antibody ELISA parameters were 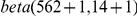 for the sensitivity and 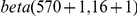 for the specificity. These were based on unpublished results provided by the ELISA manufacturer from a comparison study using VNT as a gold standard.

The model parameters were estimated using a Markov chain Monte Carlo methodology with JAGS software called from R (R core team 2009) using the Rjags package [18]. After an initial burn-in period of 200,000 samples a further 300,000 were collected from 3 MCMC chains with dispersed initial values for posterior inference. Apparent convergence of the MCMC samples was assessed by visual examination of the sample histories and calculation of the Brooks-Gelman diagnostic[19].

### Risk Factor Model

The questionnaire was designed to study foot-and-mouth disease, however, there were a number of questions relating to management factors such as contacts with other herds, wildlife, other domestic ruminants kept by the herdsman, feeding, drinking etc. that were suitable for analysis with this BVD dataset. The outcome variable of interest was whether the herd was exposed to a PI animal or not. Herds were classified as having PI exposure based on the standard “spot test” programme in the BVD eradication scheme of three or more juvenile test positives[17]. A standard univarible analysis was conducted using the ‘epicalc’ package [20] on 40 categorical herd-level variables relating to management and contacts and 3 continuous variables for herd density, area of the veterinary centre and the number of herds registered for vaccination there. Variables with a p-value of 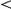
0.15 were passed onto the multivariable modelling.

The regression model was built using forward selection starting with the variables with the lowest p-values from the univariable analysis. Final model selection was based on minimizing the AIC. Variables were allowed to be re-entered as the model was refined and preference was given to exploring models incorporating biologically plausible variables.

### Mapping

The spatial distribution of within-herd prevalences, estimated using the Bayesian analysis of within-herd seroprevalence, was mapped using R software version 2.9.1 (http://cran.r-project.org/) (Packages ‘Sp’, ‘classInt’, ‘RColorBrewer’ and ‘maptools’). Herds had been geo-referenced in the initial 2000 study using hand-held GPS devices (Garmin 12, Garmin Kansas, USA). Small-scale manual jittering was applied to the plotted location of herds with similar recorded locations in order to separate plotting symbols on the published graphics. Mean estimates of the seroprevalence and time since last PI were mapped to a appropriate interval colour scales.

### Spatial correlation of BVD seroprevalence

The spatial correlation of BVD seroprevalence was examined using a permutation test. The correlation between each herd's years since last PI with its 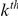 nearest neighbour was calculated over a range of 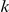 from 1 to 40. Each correlation was then compared to the distribution of correlations under a null hypothesis of no spatial correlation by randomly allocating the observed years across locations using 1000 replicates.

## Results

### Descriptive Analysis

**Figure 2:**
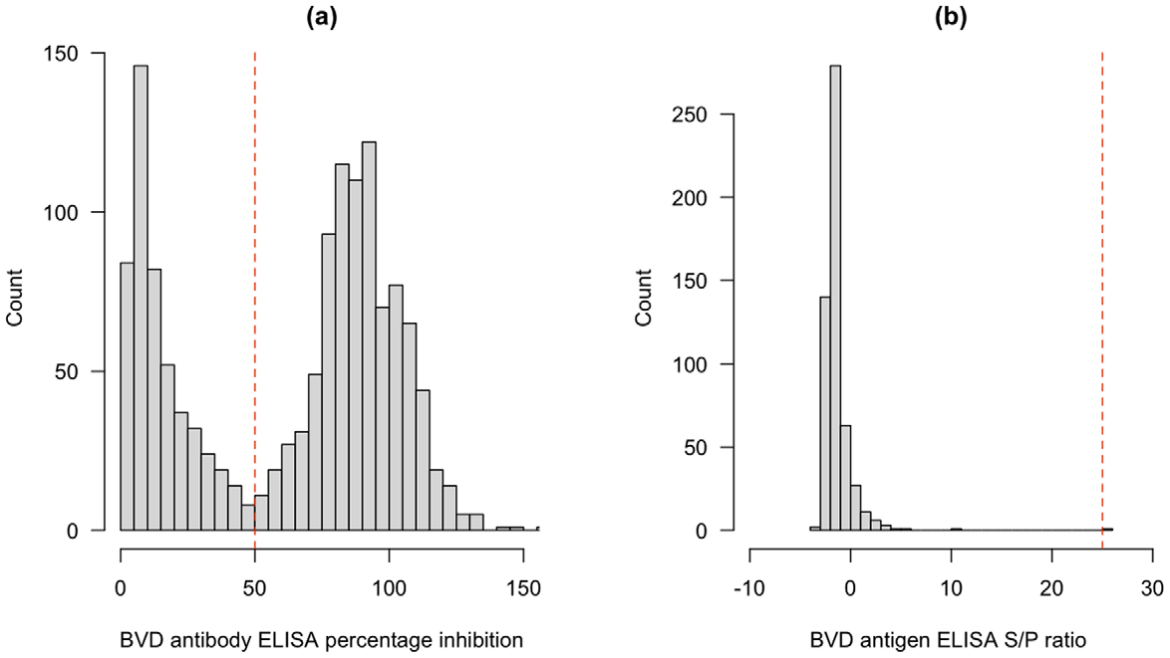
Histogram showing the number of animals against their percentage inhibition values for the BVD antibody ELISA and the sample/positive control (S/P) ratio for the antigen ELISA.

Cattle from 146 herds across the 5 administrative divisions of the Adamawa Region were sampled and screened for antibodies to pestiviruses, presumed to be to BVDV. In 28 herds fewer than 10 (5 juvenile and 5 adult) animals were made available resulting in a final sample size of 1377 animals. The median reported herd size was 36 animals (range 6 – 81). The percentage inhibition from the NS3 antibody ELISA are shown in Figure 2 (a). The percentage inhibitions show a clear bimodal distribution with the seropositive animals having a percentage inhibition 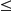 50%. The manufacturers suggest an inconclusive range between 40 and 50%. Based on these cut-offs 34.6% of the samples tested positive, 1.6% were inconclusive and 63.8% were seronegative. To maximise the specificity, a single cut-off of 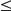
50% was used to classify each animal for subsequent analysis. A subset consisting of all 496 antibody negative juvenile samples (8 to 24 months of age) and 39 randomly selected antibody positive juvenile samples were screened with the second antigen ELISA. None of the seropositive animals were antigen positive and only 1/496 of the seronegative animals was antigen inconclusive (Figure 2 (b)).

The one sample which gave an equivocal positive result on the BVDV Ag test (S/P  = 26%) was further tested. Importation restrictions prevented attempted virus isolation from this sample so RNA was extracted and it was accordingly further tested by a previously described pan-pestivirus reverse transcriptase PCR [21] and for the presence of the recently described atypical “HoBi-like” strains [22]; no pestivirus RNA was detected by either method while an endogenous internal control (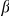-actin) signal was obtained. We therefore suspect that this was a false positive result. Given the high mortality rates expected for PIs, particularly in this harsh environment and our sampling strategy of not sampling animals less than 8 months, the lack of antigen test positives (PIs) in the sample is not surprising.

**Figure 3:**
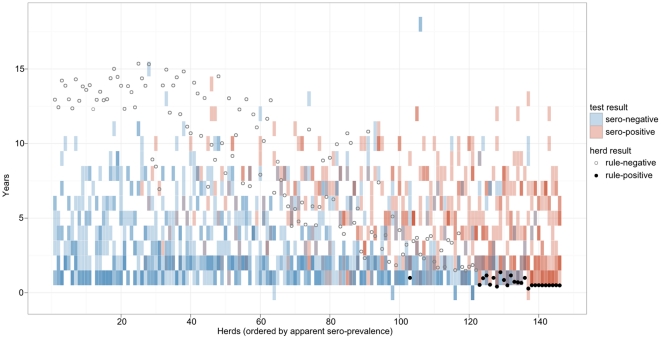
Age stratified animal-level seroprevalence based on raw test results (not adjusted for clustering within-herds or diagnostic test imperfections).

The age stratified seroprevalences are given in Figure 3 and show an increase in seroprevalence with age that appears to level at 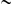
50% by 5 years of age.

### Date of the last PI in the herd

**Figure 4:**
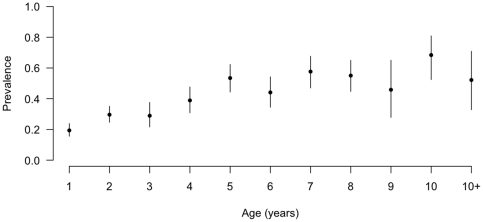
Test based classification of individual animals ordered by apparent within-herd seroprevalance and model based estimate of the date of last infection in the herd. Individual animal results are shown as tiles, blue for negative and red for positive. Multiple animals in a herd for a given age group are overlaid showing as a darker tile in blue or red. Purple tiles represent combinations of positive and negative animals in the same herd and age class. The circles show the model based estimates of time since a PI was last present in each herd. The filled circles would be classified as having a PI present using the rule of 3 or more test positive animals (SPOT test positive).

The complete categorical test results are given in Figure 4. This shows a clear progression of herds with no test positives through to those with all test positives. Overlayed on this is the putative number of years since the last PI was in the herd up to a maximum of 20 years previously. Again, there is a clear progression from herds with no seropositives that have likely not had a PI for many years through to those herds with high seroprevalences and in particular with 3 or more seropositive juvenile animals suggesting current or recent presence of a PI in the herd. 16.5% (13.7–19.2%; 95% highest posterior density interval) of herds were classed as having had a PI in the last 12 months and 26.7% (23.9–30.1; 95% HPDI) in the last 2 years. Using the spot test approach of 3 or more seropositive calves in a sample, 17.6% (11.3 – 23.9) sampled herds with juveniles were classified as having a PI which is very similar to the model based estimate for having a PI in the previous year. It is also clear from Figure 4 that there is evidence of self-clearance as there are test positive adults in herds where no juveniles were positive. The model estimated the probability of seroconversion from contact with a PI as 0.795 (0.735–0.854).

### Prevalence Estimates

**Figure 5:**
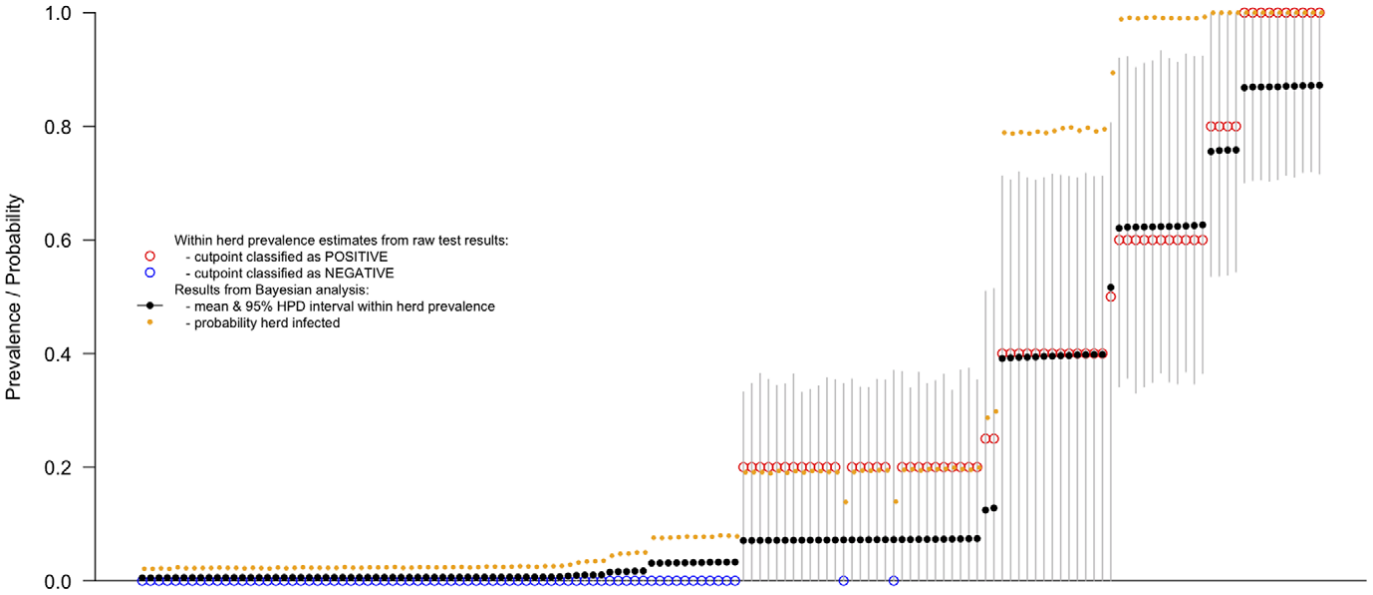
Caterpillar plots showing the estimated adjusted and unadjusted within-herd seroprevalence for each of the 142 herds based only on juvenile data, the 95% highest density intervals (HDI) for the adjusted seroprevalences and the probability that each herd is seropositive. Herds are ordered along the x axis based on the estimated within-herd seroprevalence.

Ninety two percent (HPDI: 81.8–100%) of herds were estimated to be seropositive, with a mean within-herd sero-prevalence of 38% (HPDI: 0.00–84.9%). The very wide HPDI interval reflects the wide variation in this parameter which can be more clearly seen in Figure 5 (the black dots with 95% HPDI). The individual within-herd estimates are very similar to the apparent prevalence estimates (the black dots compared to the open red and blue circles). Figure 5 also plots the probability that a herd is seropositive (orange dots).

### Mapping and Spatial Analysis

**Figure 6:**
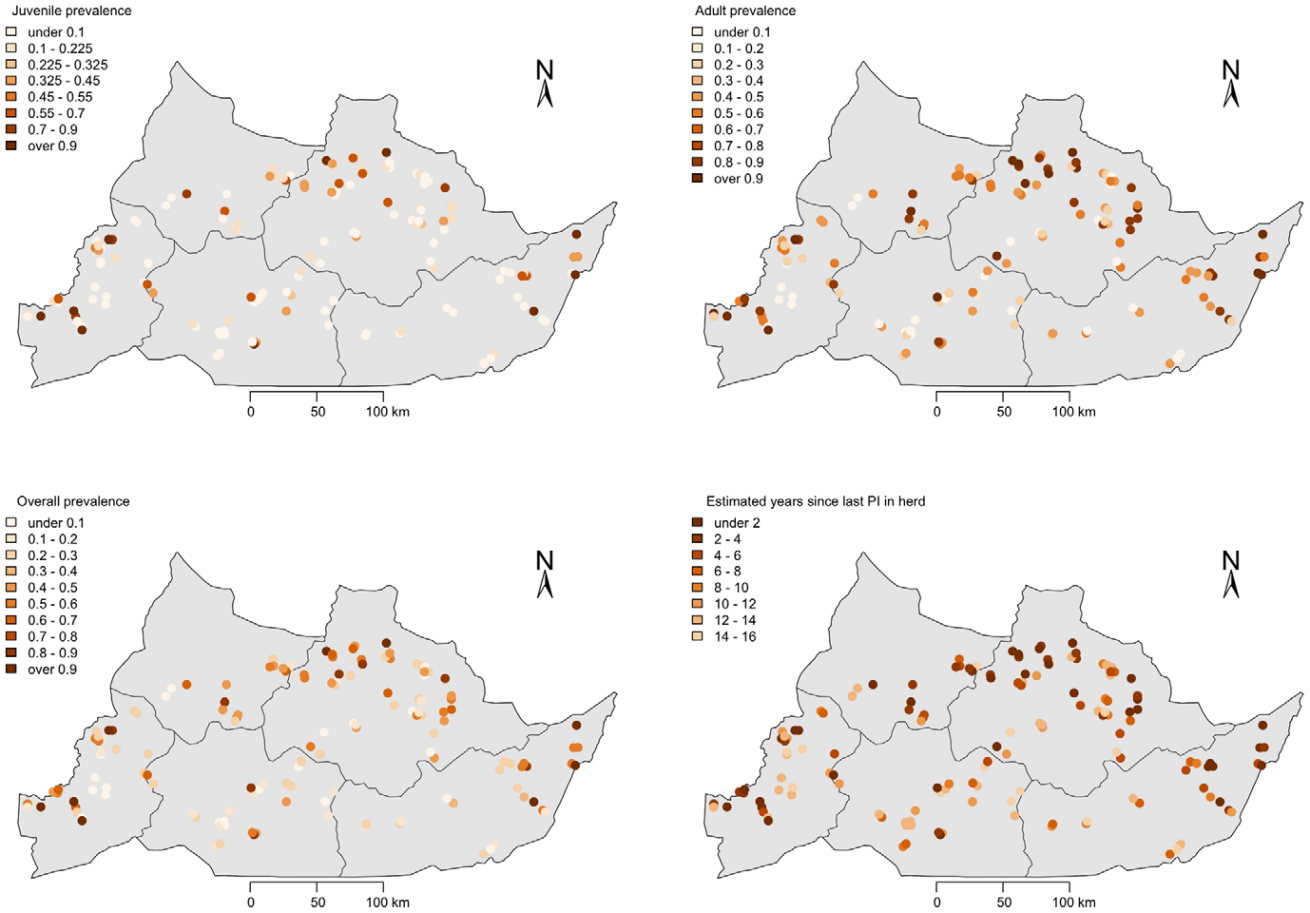
Maps of the Adamawa Region of Cameroon showing location of sampled herds and (a) within herd pestivirus seroprevalence based on juveniles only, (b) within herd pestivirus seroprevalence based on adults only, (c) overall within herd pestivirus seroprevalence and (d) the estimated years since last PI calf was in each herd.

**Figure 7:**
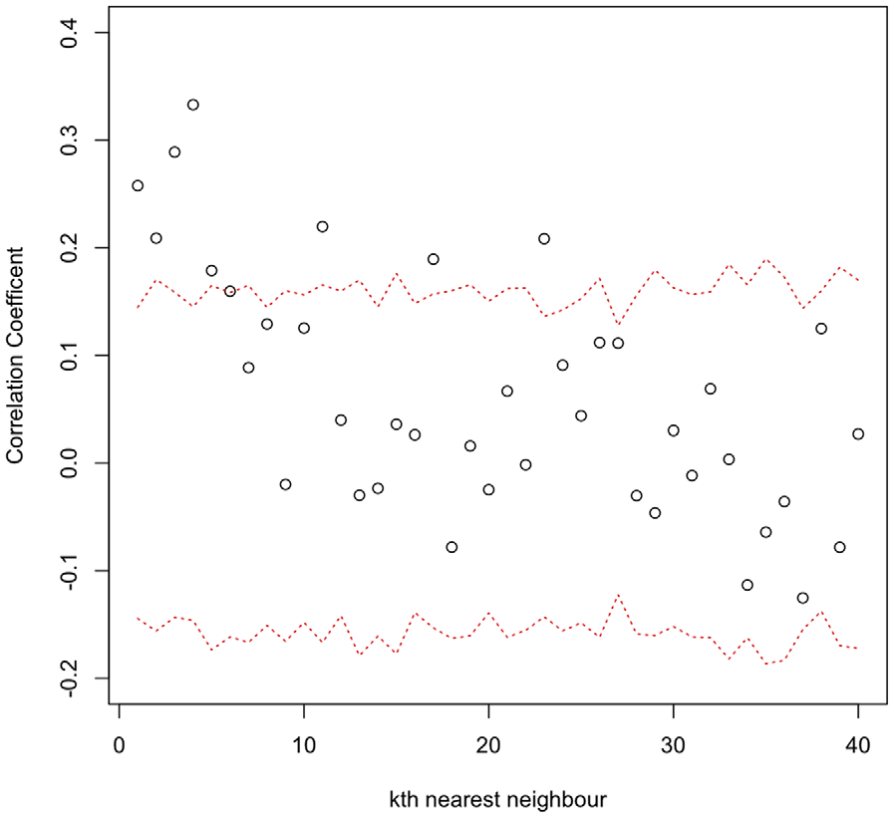
Mean correlation of the estimated years since last PI between herds and their 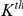 nearest neighbour and the 95% range (red dotted lines) from 1000 randomly allocated permutations.

The spatial distribution of pestivirus positive (presumed BVDV) juvenile cattle is given in Figure 6(a) and for adult cattle Figure 6 (b). The overall seroprevalence is given in Figure 6 (c) and the years since last PI is plotted in 6(d). The distribution of herds with positive juveniles is mainly across the north of the Region. The distribution of herds with positive adults is much wider spread. The clustering of time since last PI was assessed and the results of the permutation testing are given in Figure 7. This shows evidence of clustering up to the 5th nearest neighbour.

### Risk Factor Analysis for a PI in the Herd

All 142 herds with data from juvenile animals were classified as PI positive or negative based on the presence of 3 or more antibody test positives following the standard spot test approach in control programmes [23]. Forty categorical and three derived continuous variables were screened. Five variables with a p-value 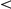
0.15 were passed forward for inclusion in a multivariable risk factor model for BVDV herd with a PI calf (Table 1). Table 2 shows a selection of models explored and their AIC. The final model is reported in Table 3 and suggests that contact with 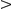
10 herds, owning goats and contact with antelopes are statistically significant risk factors. Cattle density was not retained in the model but the size of estimated area of the veterinary centre was not significant at the 5% level but suggests that larger areas have a slightly reduced risk.

**Table 1:** Univariable analysis of potential risk factors for having a persistently infected presumed bovine viral diarrhoea virus (PI) calf in the herd in the Adamawa Region of Cameroon.

Question	Variable	Levels	BVD T+	BVD T-	OR	p-value
Do you see antelope grazing near your herd?	antgrz	no	19	106	1.00	0.08
		yes	6	11	3.01	
Do you see buffalo near your herd while grazing or on transhumance?	buffevr	no	12	82	1.00	0.04
		yes	13	35	2.52	
Do you see antelope near your herd while grazing or on transhumance?	antevr	no	11	86	1.00	0.01
		yes	14	31	3.49	
Do you keep any goats?	owngt	no	16	97	1.00	0.05
		yes	9	20	2.71	
Approx. how many other herds does your herd mix with at grazing?	mxgrazct	0–10	11	78	1.00	0.02
		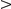 10	14	35	2.81	

**Table 2:** Comparison of herd-level logistic regression risk factor models for presumed BVD PI in herd.

Model	AIC
BVD-PI1	134.16
BVD-PIantevr	128.39
BVD-PIantevr+owngt	126.87
BVD-PIantevr+owngt+mxgrazct	122.97
BVD-PIantevr+owngt+mxgrazct+buffevr	124.71
BVD-PIantevr+owngt+mxgrazct+antgrz	124.41
BVD-PIantevr+owngt+mxgrazct+vc.area	120.47

**Table 3:** Final risk factor model for presumed BVD PI in the herd for cattle herds in the Adamawa Region of Cameroon (n = 138 herds; 4 herds with missing values for mxgrazct dropped).

Variable	Odds Ratio	p-value	95% CI
mxgrazct ( 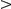 10)	2.60	0.047	1.013–6.679
owngt (yes)	2.97	0.040	1.053–8.348
antevr (yes)	4.00	0.005	1.519–10.54
vc.area ( 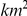 )	0.999	0.059	0.998 1.001

## Discussion

Bovine viral diarrhoea virus is globally distributed in cattle populations. Its importance in reproductive/infertility problems and respiratory disease syndromes is well established and although lacking the media impact of more acute diseases such as foot-and-mouth disease, is none the less of enormous importance financially to the cattle industry. However, very little attention has been paid to this disease in sub-Saharan Africa and its potential role as an important co-infection causing immunosuppression and the impact on other endemic diseases does not appear to have been investigated. This study is the first reported study of BVDV epidemiology in West or Central Africa.

Based on the corrected estimates using juvenile and adult data, there are high within herd and extremely high herd-level seroprevalences to pestiviruses, presumed to be BVDV, (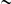
35% and 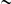
92% respectively) in the cattle population of the Adamawa Region of Cameroon. The distribution of within-herd seroprevalences is interesting as there is a very wide flat profile reflecting the range of seroprevalences within herds. This may reflect the epidemiology with herds at different stages from having a PI producing high within herd seroprevalences to those in the various stages of self-clearance[9].

There are no reports of the prevalence of PI animals in endemic settings but modelling suggests that only low prevalences are needed to maintain the virus [24]; a recent study in Switzerland suggests a PI rate of 0.64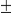 0.34% [25]. In addition, these authors note that relying on antigen detection alone risks underestimating the prevalence of PI due to test imperfections[25]. This study in Cameroon failed to detect any PI calves. Our estimate was biased by the lack of sampling in the 0–8 month old age group which was a feature of the design for the original foot-and-mouth disease study. However, using the spot test approach and our novel latent class Bayesian modelling approach of the time since last PI we have been able to identify herds that are likely to have PIs. Further, the Bayesian model allowed us to estimate the time since last PI in each individual herd and this shows quite clearly that in this setting herds are able to clear the virus naturally 4. The modelling approach and the empirical spot test approach give almost identical results supporting the model and reassuring that the spot test approach has some quantitative evidence base. This figure also shows clearly the dynamics at the population level of self-clearance of pestivuses (presumed to be BVDV) from herds. This approach could be extended to look at the rates of self-clearance and the potential contribution that could make to control in this setting compared to temperate cattle populations exposed to fewer pathogens.

The apparent self-clearance of virus form many herds is interesting and needs further investigation in a longitudinal study. From the literature [26] important factors in promoting self-clearance are reported as early removing of PI animals, few animals in early gestation exposed to PI, lack of breeding synchronisation, smaller herds and contact rates. In this setting many of these factors may favour clearance with small herds, lack of seasonal breeding etc. but this is an issue that needs further study and modelling.

The cattle rearing in the Region is very traditional and extensive with herds communally grazed and 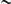
47% moving on transhumance each dry season [13]. There is no set seasonal calving and so dams will be at varying stages of gestation providing sufficient animals at the critical stage to infect and produce PI calves to maintain the disease. This is comparable to other recent studies in BVDV endemic countries such as Jordan [27] with reported animal and herd-level seroprevalence of 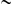
32% and 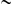
81% respectively. This contrasts with, for example, Canada where reported seroprevalences are 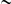
28% and 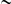
9% respectively in unvaccinated animals and herds [28], the difference in herd-level seroprevalence likely reflecting the effects of control.

The regression modelling identified contact with more than 10 herds per day at grazing (another proxy for higher density), owning goats and contact with antelope as statistically significant risk factors. This is consistent with the known epidemiology, and small ruminants [29] and deer have been highlighted as potential reservoirs in eradication campaigns. Buffalo and eland in particular have been suggested as potential reservoirs in Africa [7], [30] although this is largely based on indirect serological evidence. Similar types of contact have been reported elsewhere such as common use of pasture in Norway [31]. This may have important implications for control if the virus is genuinely maintained in these free roaming wildlife populations in Africa. However, in Scandinavia where control as been successful this has been achieved without need to control the virus in wildlife and this may be the case in Africa.

The spatial distribution of recently exposed herds (i.e. in the last 2 years) based on juvenile seropositives to pestivruses is very suggestive of a clustering of transmission in the higher cattle density areas in the arc across the north of the Region. This was not borne out by the regression analysis that did not find any statistical association with cattle density. However, the physical area of the polygon (vc.area), putatively the catchment area for the veterinary centre, was protective and though not statistically significant, did improve the fit of the model based on the AIC. This suggests that veterinary centres covering larger areas have lower risk and is possibly a proxy for lower density as well. This may be partly due to the rather crude way we have estimated the cattle density using the vorooni tesellation. The number of seropositive herds based on the juveniles may be an underestimate due to the small within herd sample. However, the sample size was considered sufficient to identify herds with PIs due to the very high transmission rates from PIs. The distribution of herds by the years since last PI in the herd (Figure 5d) based on the model estimates, appears to largely agree that most recent transmission is in this arc. In addition the formal cluster analysis strongly supports the hypothesis that higher seroprevalence herds tend to cluster together after accounting for any underlying clustering in the locations of herds. This would suggest potential epidemiology dominated by local direct contact rather than the markets consistent with the regression model. Further dynamic transmission modelling and ideally molecular typing would be needed to investigate the potential contributions of the different contact types and animal movements to and from markets.

Control of BVD has been achieved in countries by detection of infected herds and removal of PI animals, supported by vaccination or biosecurity measures, however, in developing countries without the veterinary infrastructure this is unlikely to be achieved in the near future. In developed countries there is general agreement about the cost/benefits of control [15], [32], [33]. There may be similar benefits for developing countries but these need to be reliably estimated. This has important implications for production as endemic BVD causes reproductive and calf losses with the potential for secondary losses. Control in an African setting would be challenging given the levels of animal movements and lack of controls of movement and trade in the region. Furthermore, its importance in co-infections with such diseases as foot-and-mouth disease, *Mycobacterium spp*. and infectious respiratory disease have yet to be fully investigated in this setting and control may have potential additional benefits beyond the direct benefits of control of BVDV itself.

The Bayesian modelling approach applied here proved useful as it allowed the incorporation of the diagnostic test sensitivity and specificity, the uncertainties in these parameters and the study design features. In addition it permits the estimation of latent parameters such as the time since last PI was in the herd. One of the clear implications of this estimation approach is that the within-herd sample sizes were small for the purpose of estimating within-herd seroprevalences (they were originally designed to estimate within-herd FMD prevalence). However, the Bayesian approach has allowed unbiased estimates of seroprevelance from a design that was not intended for studying this disease, allowing maximum information to be extracted from the survey and banked material and provided robust estimates for these infections. The very wide range of within-herd seroprevalences is problematic when design a sampling strategy as conventional approaches require a single point estimate on which to base the calculation. However, as we have observed, there are herds with low seroprevalence where too few animals will have been sampled to be achieve required confidence, given the test performance, that the herd is seronegative. This will underestimate herd-level seroprevalence.

In conclusion, this study has demonstrated very high herd seroprevalences to pestiviruses, which we have assumed to be mainly due to BVDV, and we estimate 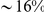 of herds had PI animals although none were detected probably due to the sampling strategy. We have also shown that there is self-clearance of the virus from previously infected herds. These will be extremely useful estimates in modelling potential control and eradication options for settings. There is also a clear need to look at the role of BVDV in co-infections in the African setting to fully understand its importance and evaluate the need and importance for it control.
